# Postoperative Care and Functional Recovery After Laparoscopic Sleeve Gastrectomy vs. Laparoscopic Roux-en-Y Gastric Bypass Among Patients Under ERAS Protocol

**DOI:** 10.1007/s11695-017-2964-3

**Published:** 2017-10-23

**Authors:** Piotr Major, Tomasz Stefura, Piotr Małczak, Michał Wysocki, Jan Witowski, Jan Kulawik, Mateusz Wierdak, Magdalena Pisarska, Michał Pędziwiatr, Andrzej Budzyński

**Affiliations:** 1 2nd Department of General Surgery, Jagiellonian University Medical College, Kopernika 21 St., 31-501 Kraków, Poland; 2 Centre for Research, Training and Innovation in Surgery (CERTAIN Surgery), Krakow, Poland; 3 Students’ Scientific Group at 2nd Department of Surgery, JUMC, Krakow, Poland

**Keywords:** Bariatric surgery, Sleeve gastrectomy, Gastric bypass, Postoperative care, ERAS

## Abstract

**Background:**

The most commonly performed bariatric procedures are laparoscopic sleeve gastrectomy (LSG) and laparoscopic Roux-en-Y gastric bypass (LRYGB). There are major differences between LSG and LRYGB during postoperative period. Optimization of the postoperative care may be achieved by using enhanced recovery after surgery (ERAS) protocol, which allows earlier functional recovery.

**Purpose:**

The aim was to assess differences in the course of postoperative care conducted in accordance with ERAS protocol among patients after LSG and LRYGB.

**Material and Methods:**

Data concerning patients treated for morbid obesity were prospectively gathered in one academic center. Patients were divided into two groups: LSG (*n* = 364, 63.41%) and LRYGB (*n* = 210, 36.59%). Multiple factors were used as endpoints to determine the influence of the type of bariatric procedure on postoperative course.

**Results:**

The rate of postoperative nausea and vomiting and incidence of intravenous fluid administration during the operation was higher in LSG group. LRYGB patients were able to tolerate higher oral fluid intake volumes during the first and the second postoperative day. Mean diuresis during the second and the third postoperative day was significantly higher in LRYGB group. Administration of diuretics and painkillers was comparable between groups, while the risk of fever after the operation was higher in LRYGB group. Mean length of stay was higher in LSG group (LRYGB vs. LSG, 3.46 days ± 1.58 vs. 3.64 days ± 4.41, *p* = 0.039).

**Conclusions:**

In our opinion, postoperative treatment after LSG requires more supervision and longer time until functional recovery is achieved.

## Introduction

Bariatric surgery seems to be the most effective treatment for obesity and obesity-related metabolic comorbidities [1]. The most commonly performed bariatric procedures worldwide are laparoscopic sleeve gastrectomy (LSG) and laparoscopic Roux-en-Y gastric bypass (LRYGB) [2, 3]. Enhanced recovery after surgery (ERAS) protocol during the course of postoperative care includes avoiding use of catheters and intra-abdominal drains, prophylactic use of antithrombotic medications, early mobilization, early enteral feeding, and multimodal postoperative analgesia [4, 5]. Perioperative care carried out in accordance to ERAS protocol seems to be a safe and feasible method for both operations, which allows to reduce length of hospital stay and readmission rates without influencing morbidity [6, 7]. Contrary to popular opinion, the main benefit from implementing the protocol is not the ability to discharge the patient as early as it is possible, but as soon as they reach full functional recovery. High demand for bariatric operations creates tendency to perform them as outpatient procedures. Optimization of the postoperative period according to ERAS protocol is a key factor for this approach [8, 9]. Major differences between patients after LSG and LRYGB during postoperative course are not well known. Achieving early recovery of gastrointestinal function seems to be significant factor contributing to decreasing discomfort or risk of prolonged hospital stay after abdominal surgery [10]. Although there are plenty of studies comparing early and late postoperative outcomes of those two procedures, none so far focused on postoperative care, especially with ERAS approach.

## Purpose

The aim was to assess the differences in postoperative course among patients treated according to ERAS protocol submitted to LSG and LRYGB.

## Material and Methods

### Study Design

**Fig. 1:**
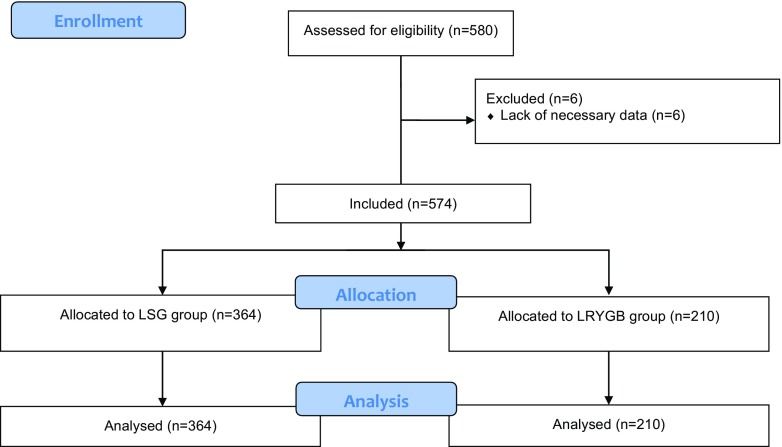
Study flowchart

Data concerning bariatric patients treated for morbid obesity in one academic center were prospectively gathered. Recommendations of the Metabolic and Bariatric Surgery Section of the Polish Surgical Society were used as indication for surgery, that is body mass index (BMI) ≥ 35 kg/m^2^ with obesity-related comorbidities or BMI ≥ 40 kg/m^2^ [11, 12]. Inclusion criteria for this study were informed consent to participate in the study, meeting the eligibility criteria for bariatric treatment, either for LSG or LRYGB. We excluded patients with insufficient data (Fig. 1). Study was designed and described according to all STROBE checklist points for observational studies [13].

Data collection was performed by authors, who were also directly involved in treatment process. Database included demographic characteristics and factors related to the surgery. Features describing patient profile included age, sex, maximal preoperative BMI, BMI on a day of operation, American Society of Anesthesiologists (ASA) class, and main comorbidities (cardiovascular diseases, arterial hypertension, obstructive sleep apnea syndrome, diabetes mellitus type 2). Perioperative variables were type of procedure, operative time, intraoperative adverse events, postoperative complications, length of hospital stay (LOS), readmissions, reoperations, fever, postoperative nausea and vomiting, stool passage after surgery during the hospitalization, diuretics management, painkillers management, and data concerning postoperative fluid management (intravenous fluid administration, oral fluid intake, and diuresis) during the operation day and first three consecutive postoperative days. Patients were divided into two groups: LSG and LRYGB. An intraoperative adverse event was defined as any iatrogenic harmful event occurring during operation, which had not derived from standard LSG or LRYGB technique. We defined postoperative complications as adverse events occurring within 1 year of the procedure. Rhabdomyolysis was defined as elevated levels of creatinine phosphokinase (CPK > 1000 IU/l) with coexisting increase of myoglobin level. Gastrointestinal leakage was defined as leakage from the GI tract clinically diagnosed and confirmed with radiological examination. Postoperative hemorrhage was defined as a significant drop in hemoglobin count with either clinically demonstrated hemorrhage requiring reoperation or need of erythrocyte transfusion. LOS was defined as period from admission to discharge, based on number of nights spent in hospital. All patients were admitted to hospital 1 day prior to surgery, so if the patient would be discharged on the day of surgery, his LOS would be 1 day.

### Treatment Protocol

In order to minimize bias, patients were treated in accordance with enhanced recovery after surgery (ERAS) pathway, including preoperative, intraoperative, and postoperative interventions [6, 14, 15]. During the preoperative period, patients were appropriately counseled. The health status of all patients was assessed with particular emphasis on incidence of type 2 diabetes mellitus, hypertension, obstructive sleep apnea, and gastroesophageal reflux disease (GERD). Incidence of GERD was assessed during history taking by a direct question and questions concerning taking proton-pump inhibitors. Preoperative consultations included also routine endoscopy of the upper gastrointestinal tract with assessment of hiatal hernia and esophageal, gastric, or duodenal mucosa pathology. In case of a large hiatal hernia with inflammatory lesions in esophageal mucosa, patients were treated with pantoprazole and qualified for LRYGB. If hiatal hernia did not coexist with inflammatory lesions in esophageal mucosa or clinical symptoms of GERD requiring pharmacotherapy, we suggested LSG with possible simultaneous cruroplasty in case of large gap in the diaphragm. Final decision was made by patients. During endoscopy, gastric mucosa tissue biopsy sample was obtained to perform rapid urease test for *Helicobacter pylori* infection (*Campylobacter-*like organism (CLO) test). In case of positive result of CLO test, eradication was conducted accordingly to guidelines from the Polish Society of Gastroenterology for the diagnosis and treatment of *Helicobacter pylori* infection [16]. Nutritional intervention included high-protein and high-carbohydrate drinks. General anesthesia, obligatory in laparoscopic surgery, was conducted accordingly to the optimized bariatric anesthetic protocol with the use of multimodal analgesics. Patients undergoing LRYGB and LSG did not require standard usage of the nasogastric tubes and intra-abdominal drains. Postoperative pain management did not include standard usage of opioids. Enoxaparin used for antithrombotic prophylaxis was administered for 14 consecutive postoperative days. All patients received routinely pantoprazole (40 mg/day) for 30 consecutive postoperative days or longer in case of postoperative incidence of GERD symptoms. After surgery, patients were mobilized and had enteral feeding introduced early. Our target is to not use intravenous fluids in postoperative period routinely. Fluids were given only if we observed absence of sufficient functional recovery: vomiting, insufficient oral fluid intake (less than 500 ml until 4:00 pm), insufficient diuresis, or biochemical symptoms of rhabdomyolysis expressed by the high blood level of myoglobin, CPK, and low GFR (glomerular filtration rate). Every bariatric patient in our center is scheduled to have three follow-up appointments: 2 weeks after discharge, 1 month after discharge, and 3 months after discharge.

### Surgical Technique

Surgical techniques for LSG and LRYGB were standardized [17]. Veress needle was used to achieve pneumoperitoneum (15 mmHg). Routine procedure required insertion of four trocars during LSG and five trocars during LRYGB. A sealer/divider or ultrasonic shears were used for a dissection and coagulation (LigaSure Atlas™, Covidien or SonoSurg™, Olympus). A 34-French gastric bougie inserted into the stomach along the lesser curvature was used to calibrate the gastric sleeve. Gastrectomy started 4–5 cm proximal to the pylorus with continuously applied linear staplers, starting with two firings of 60 mm, Ethicon Echelon EndoFlex with gold cartridges (3.8 mm open stapler height, 1.8 mm closed stapler height), then continued with blue cartridges (3.6 mm open stapler height, 1.5 mm closed stapler height) straight to the angle of His. Stapler line was reinforced by a running 3-0 PDS suture. Resected portion of the stomach was removed from the peritoneal cavity through the left flank trocar site during LSG. LRYGB required creation of a pouch by one horizontal 45-mm stapler followed by vertical stapling toward the angle of His, until the pouch was totally separated from the rest of the stomach. Gastrojejunal anastomosis was created with a linear stapler Ethicon Echelon EndoFlex (45 mm, with blue cartridges, open staple height 3.5 mm, closed staple height 1.5 mm) with hand-sewn closure of the remaining defect (3/0 Vicryl, Ethicon). The length of alimentary and enzymatic limb was standardized in all patients, respectively, 150 and 100 cm. Jejunojejunal anastomosis was created using a linear stapler Ethicon Echelon EndoFlex (45 mm, with white cartridge, open staple height 2.5 mm, closed staple height 1 mm). Petersen’s defect was not routinely closed as prevention for internal hernias. A routine 10/12 mm port sites closure was performed to prevent herniation.

### Analysis of Endpoints

Our primary endpoints were factors determining the influence of the type of bariatric procedure on postoperative functional recovery:

- Postoperative nausea and vomiting (PONV)
- Stool passage
- Intravenous fluid administration
- Oral fluid intake during hospitalization
- Diuresis during hospitalization
- Diuretics management
- Extra painkillers management
- Postoperative fever
- Length of hospitalization (LOS)
- Readmissions

Secondary endpoint was analysis of operative outcomes influenced by the type of bariatric procedure:

- Operative time
- Mean IV fluid administration during the operation
- Intraoperative adverse events
- Postoperative complications
- Reoperations

### Statistical Analysis

All data were analyzed with Statistica version 12.0 PL (StatSoft Inc., Tulsa, OK, USA). The results are presented as mean standard deviation (SD), median and interquartile range (IQR), and odds ratio (OR) with 95% confidence intervals (CI) when appropriate. The study of categorical variables used the chi-square test of independence. Shapiro-Wilk test was used to check for normal distribution of data, and the Student *t* test was used for normally distributed quantitative data. For non-normally distributed quantitative variables, the Mann-Whitney *U* test was used. Influence of the type of bariatric procedure on postoperative complications, gastrointestinal leakage, gastrointestinal stricture, postoperative hemorrhage, wound infection, pneumonia, reoperation, readmission, fever, PONV, stool passage, extra pain killer management, diuretics management, and incidence of intravenous fluid administration during the operation day and first to third postoperative day rates was analyzed in univariate logistic regression models. *p* value < 0.05 was considered statistically significant. Statistical power analysis was performed with power and sample size calculator for odds ratio: equality available from: http://powerandsamplesize.com/Calculators based on method by Chow et al. [18].

Five hundred eighty patients were treated for morbid obesity at tertiary referral academic institution from April 2009 to November 2016. Five hundred seventy-four patients met inclusion criteria and underwent LSG or LRYGB (362 females, 212 males, mean age 42.77 ± 11 years). Three hundred sixty-four (63.41%) patients underwent LSG (245 females, 119 males, mean age 40.88 ± 11.1 years) and 210 (36.59%) patients underwent LRYGB (117 females, 93 males, mean age 46.06 ± 10.08 years) (Fig. 1). Median maximal preoperative BMI and BMI on a day of operation were significantly higher in LRYGB group. Higher ASA classes were more often present in LRYGB group. Patients in LRYGB group presented significantly greater rates of comorbidities, including cardiovascular diseases (CVD), arterial hypertension (HTN), and diabetes mellitus (DM). Rates of obstructive sleep apnea (RD) were similar in bath groups (Table 1).

**Table 1:** Patients and groups baseline characteristics

Parameter		All patients	LSG	LRYGB	*p*
*n*		574 (100%)	364 (63.41%)	210 (36.59%)	–
Females, *n* (%)		362 (63.07%)	245 (67.31%)	117 (55.71%)	*0.005*
Males, *n* (%)		212 (36.93%)	119 (32.69%)	93 (44.29%)	
Mean age (years ± SD)		42.77 ± 11	40.88 ± 11.1	46.06 ± 10.08	*< 0.001*
Median maximal preoperative BMI, kg/m^2^ (IQR)		46.87 (42.93–51.63)	46.04 (42.81–50.61)	48.26 (43.42–53.71)	*0.009*
Median BMI on a day of operation, kg/m^2^ (IQR)		45.28 (41.45–50.03)	44.86 (41.31–48.9)	46.06 (41.81–51.64)	*0.049*
Preoperative BMI loss, kg/m^2^ (IQR)		0.99 (0–2.47)	0.78 (0–2.26)	1.38 (0–2.81)	*0.028*
Median ASA (IQR)		2.00 (2.00–3.00)	2.00 (2.00–2.00)	2.00 (2.00–3.00)	**<** *0.001*
ASA class, *n* (%)	1	15 (2.61%)	14 (3.85%)	1 (0.48%)	**<** *0.001*
	2	386 (67.25%)	262 (71.98%)	124 59.05(%)	
	3	157 (27.35%)	84 (23.08%)	73 (34.76%)	
Cardiovascular diseases, *n* (%)		107 (18.64%)	53 (14.56%)	54 (25.71%)	**<** *0.001*
Hypertension, *n* (%)		395 (68.82%)	229 (62.91%)	166 (79.05%)	**<** *0.001*
Diabetes mellitus, *n* (%)		198 (34.49%)	88 (24.18%)	110 (52.38%)	**<** *0.001*
Obstructive sleep apnoea, *n* (%)		45 (7.84%)	29 (7.97%)	16 (7.62%)	0.881

## Results

Our study group reveals higher risk of PONV in LSG group (LRYGB vs. LSG, OR 0.16, CI 0.05–0.54, *p* = 0.003). Amount of intravenous fluid administration during the operation day was significantly higher in LSG group (LRYGB vs. LSG, OR 0.60, CI 0.41–0.89, *p* = 0.01). Volume of intravenous fluid administration was comparable in both groups during the first, second, and third postoperative day. There was no significant difference in oral fluid intake during the operation day. Oral fluid intake during the first (LRYGB vs. LSG, 1532.94 ml ± 575.4 vs. 1213.62 ml ± 689.46, *p* < 0.001) and the second (LRYGB vs. LSG, 1978.23 ml ± 776.86 vs. 1456.77 ml ± 743.38, *p* < 0.001) postoperative day was significantly higher among patients who underwent LRYGB. However, there was no difference in oral fluid intake during the third postoperative day. Although mean diuresis during the operation day and first postoperative day was similar in both groups, mean diuresis during the second (LRYGB vs. LSG, 2683.88 ml ± 916.06 vs. 2369.26 ml ± 767.15, *p* = 0.001) and the third (LRYGB vs. LSG, 2662.03 ml ± 798.28 vs. 2346.71 ml ± 848.58, *p* = 0.034) postoperative day was significantly higher in LRYGB group. The risk of postoperative fever was higher in LRYGB group (LRYGB vs. LSG, OR 1.93, CI 1.22–3.05, *p* = 0.005). Administration of diuretics (LRYGB vs. LSG, OR 1.25, CI 0.89–1.77, *p* = 0.198) and painkillers (LRYGB vs. LSG, OR 1.03, CI 0.72–1.47, *p* = 0.880) was comparable in both groups. Rates of stool passage during hospitalization were similar in both groups (LRYGB vs. LSG, OR 1.34, CI 0.95–1.88, *p* = 0,094). Mean LOS was significantly higher in LSG group (LRYGB vs. LSG, 3.46 days ± 1.58 vs. 3.64 days ± 4.41, *p* = 0.039). Readmission rates were not influenced by the operation (LRYGB vs. LSG, OR 1.70, CI 0.87–3.32, *p* = 0.119) (Tables 2 and 3).

**Table 2:** Postoperative course and functional recovery in groups LSG vs. LRYGB

	Total	LSG	LRYGB	*p*
*n*	574 (100%)	364 (63.41%)	210 (36.59%)	–
PONV	33 (5.75%)	30 (8.24%)	3 (1.43%)	*< 0.001*
Stool passage	277 (48.26%)	166 (45.6%)	111 (52.86%)	0.119
Mean IV fluid administration during the operation day (ml ± SD)	807.08 ± 439.53	801.96 ± 404.61	819.79 ± 520.85	0.811
Mean IV fluid administration during the first post-op day (ml ± SD)	1715.58 ± 1031.19	1632.05 ± 906.08	1846.94 ± 1194.08	0.287
Mean IV fluid administration during the second post-op day (ml ± SD)	1537.03 ± 885.84	1499.12 ± 899.76	1600.58 ± 864.84	0.276
Mean IV fluid administration during the third post-op day (ml ± SD)	1473.38 ± 720.0–0	1592.56 ± 645.94	1328.12 ± 787.24	0.125
IV fluid administration during the operation day	171 (29.79%)	122 (33.52%)	49 (23.33%)	*0.003*
IV fluid administration during the first post-op day	319 (55.57%)	195 (53.57%)	124 (59.05%)	0.340
IV fluid administration during the second post-op day	182 (31.71%)	114 (31.32%)	68 (32.38%)	0.926
IV fluid administration during the third post-op day	71 (12.37%)	39 (10.71%)	32 (15.24%)	0.518
Mean oral fluid intake during the operation day (ml ± SD)	427.05 ± 432.1	428.55 ± 418.55	424.56 ± 454.96	0.921
Mean oral fluid intake during the first post-op day (ml ± SD)	1333.46 ± 666.58	1213.62 ± 689.46	1532.94 ± 575.4	*< 0.001*
Mean oral fluid intake during the second post-op day (ml ± SD)	1676.7 ± 799.15	1456.77 ± 743.38	1978.23 ± 776.86	*< 0.001*
Mean oral fluid intake during the third post-op day (ml ± SD)	1781.68 ± 683.55	1763.15 ± 751.90	1807.27 ± 581.77	0.717
Mean diuresis during the operation day (ml ± SD)	2269.82 ± 814.85	2253.61 ± 768.47	2296.57 ± 887.71	0.573
Mean diuresis during the first post-op day (ml ± SD)	2516.42 ± 802.29	2497.46 ± 781.06	2548.02 ± 837.79	0.508
Mean diuresis during the second post-op day (ml ± SD)	2497.87 ± 844.1	2369.26 ± 767.15	2683.88 ± 916.06	*0.001*
Mean diuresis during the third post-op day (ml ± SD)	2477.69 ± 839.53	2346.71 ± 848.58	2662.03 ± 798.28	*0.034*
Diuretic management	327 (56.97%)	200 (54.95%)	127 (60.48%)	0.281
Extra painkiller management	200 (34.84%)	126 (34.62%)	74 (35.24%)	0.949
Postoperative fever	88 (15.33%)	44 (12.09%)	44 (20.95%)	*0.006*
Median LOS (days, IQR)	3 (2–4)	3 (2–4)	3 (2–4)	*0.039*
Mean LOS (days ± SD)	3.57 ± 3.6	3.64 ± 4.41	3.46 ± 1.58	
Readmissions	37 (6.45%)	19 (5.22%)	18 (8.57%)	0.163

**Table 3:** Influence of LRYGB vs. LSG group on OR of primary endpoints

Event	OR	95% CI	*p*	Test power, %
PONV	0.16	0.05–0.54	*0.003*	*96.36%*
Stool passage	1.34	0.95–1.88	0.094	55.95%
IV fluid administration during the operation day	0.60	0.41–0.89	*0.010*	*89.65%*
IV fluid administration during the first post-op day	1.41	0.99–2.01	0.054	36.06%
IV fluid administration during the second post-op day	1.05	0.73–1.51	0.792	6.3%
IV fluid administration during the third post-op day	1.5	0.9–2.47	0.115	51.23%
Diuretic management	1.25	0.89–1.77	0.198	36.82%
Extra painkiller management	1.03	0.72–1.47	0.880	5.44%
Fever	1.93	1.22–3.05	*0.005*	*94.22%*
Readmissions	1.70	0.87–3.32	0.119	5.15%

Operative time was significantly longer in LRYGB group [LSG vs. LRYGB, 100 (80–120) vs. 140 (110–180), *p* < 0.001]. Mean volume of intraoperative fluids administration was significantly higher in LRYGB group (LRYGB vs. LSG, 1552.47 ml ± 537.24 vs. 1336.05 ml ± 501.87, *p* < 0.001). Incidence of intraoperative adverse events was comparable in both groups. Rates of general postoperative complications and specific complications, including gastrointestinal leakage, gastrointestinal stricture, postoperative, wound infection, and pneumonia, were not linked the type of bariatric procedure performed. There were no significant differences in severity of postoperative complications, assessed in accordance with Clavien-Dindo classification, between LSG and LRYGB groups [19]. We did not observe any significant relation between the type of procedure and increased risk of reoperation (Tables 4 and 5).

**Table 4:** Early postoperative outcomes in groups LSG vs. LRYGB

	Total	LSG	LRYGB	*p*
*n*	574 (100%)	364 (63.41%)	210 (36.59%)	–
Mean operative time (min ± SD)	121.07 ± 47.86	105.99 ± 39.95	146.23 ± 49.45	**<** *0.001*
Median operative time (min, IQR)	115 (87.5–150)	100 (80–120)	140 (110–180)	
Mean IV fluid administration during the operation (ml ± SD)	1416.56 ± 525.32	1336.05 ± 501.87	1552.47 ± 537.24	**<** *0.001*
Intraoperative adverse events	24 (4.18%)	12 (3.3%)	12 (5.71%)	0.163
Postoperative complications	44 (7.67%)	26 (7.14%)	18 (8.57%)	0.542
Clavien-Dindo I–II	27 (4.7%)	18 (4.95%)	9 (4.29%)	0.719
Clavien-Dindo III–V	17 (2.96%)	8 (2.2%)	9 (4.29%)	0.155
Biochemical rhabdomyolysis	10 (1.74%)	3 (0.82%)	7 (3.33%)	0.916
Gastrointestinal leakage	6 (1.05%)	4 (1.1%)	2 (0.95%)	0.868
Gastrointestinal stricture	9 (1.57%)	7 (1.92%)	2 (0.95%)	0.367
Postoperative hemorrhage	7 (1.22%)	3 (0.82%)	4 (1.9%)	0.256
Wound infection	2 (0.35%)	1 (0.27%)	1 (0.48%)	0.759
Pneumonia	6 (1.05%)	2 (0.55%)	4 (1.9%)	0.124
Reoperations	9 (1.57%)	5 (1.37%)	4 (1.9%)	0.317

**Table 5:** Influence of LRYGB vs. LSG group on OR of secondary endpoints

Event	OR	95% CI	*p*	Test power, %
Intraoperative adverse events	1.78	0.78–4.03	0.169	40.88%
Postoperative complications	1.22	0.65–2.28	0.536	12.25%
Clavien-Dindo I–II	0.86	0.38–1.95	0.720	7.41%
Clavien-Dindo III–V	1.99	0.76–5.25	0.163	42.04%
Biochemical rhabdomyolysis	4.15	1.06–16.22	*0.040*	*73.11%*
Gastrointestinal leakage	0.87	1.57–4.77	0.868	5.56%
Gastrointestinal stricture	0.49	0.10–2.38	0.377	20.01%
Postoperative hemorrhage	2.34	0.52–10.54	0.270	28.55%
Wound infection	1.74	0.11–27.92	0.697	8.09%
Pneumonia	3.51	0.64–19.35	0.149	28.55%
Reoperations	1.39	0.37–5.25	0.623	9.52%

## Discussion

Surgery-induced weight loss seems to be highly effective in treatment of obesity and obesity-related comorbidities [20], and LRYGB and LSG are currently the most frequently chosen bariatric procedures [21]. In our study, rates of intraoperative adverse events were comparable between groups. The median operating time was notably longer in LRYGB group, which might be related to higher volume of intraoperative fluids. Moreover, these results seem to be consistent with previously published studies [22–27]. This may have resulted in a large difference in incidence of necessity to administrate IV fluids postoperatively on the day of operation. Our data set revealed also a 5.48% higher rate of IV fluid administration on the first postoperative day in LRYGB group, but the difference was not statistically significant. Analysis of postoperative complication rates between LRYGB and LSG, according to our results and randomized clinical trial by Peterli et al., did not reveal significant differences [23]. Patients achieve similar bariatric effect both in terms of weight loss and remission of obesity-associated comorbidities, regardless of the approach [28]. Nevertheless, there are major technical differences between those two operations, and we assume that it may result in inequalities during the postoperative course and functional recovery.

Perioperative care according to the principles of ERAS protocol, which is also applied in our center, was proved to be feasible, effective, and enabling early discharge in bariatric surgery [14, 29, 30]. Barreca and colleagues published an article stating that implementation of ERAS protocol among bariatric patients led to > 40% of patients being discharged within 24 h from the operation [31]. The criteria for discharge defined in ERAS protocol include full mobilization, oral ingestion of an appropriate amount of liquid nutrition without the need for intravenous administration, appropriate diuresis, adequate post-discharge support (e.g., a family member), and the lack of objective contraindications for discharge [14]. All those parameters affect the time to reach functional recovery which in our opinion is a priority over shorter LOS. Implementation of ERAS protocol allows earlier functional recovery and reduction of the profound stress response after surgery [32].

Most of previous publications comparing LRYGB and LSG focused on bariatric effect of the operations and resolution of obesity-related comorbidities. Our study is the first one that compares early postoperative course and functional recovery of the patients undergoing LSG and LRYGB under ERAS protocol. For patients undergoing surgery in this approach, first days after the surgery are crucial for maintaining homeostasis via controlling metabolism, administration of fluids, and the support of the return of key functions (i.e., improvements in cardiopulmonary function, earlier return of bowel function, earlier resumption of normal activities) [32, 33]. Primary goal is to achieve early functional recovery (lack of nausea, vomiting, or fever), which is not necessarily associated with shorter LOS. Thus, our primary endpoints focused on these parameters.

PONV is a frequent symptom of delayed functional recovery. Nausea, vomiting, and dehydration are responsible for 17.5% of indications for emergency department returns, readmissions, and reoperations after bariatric surgery [34]. Post-bariatric nausea and vomiting are directly correlated with the length of the surgery; they also appear more often in female patients, non-smokers, and those with prior history of vomiting or motion sickness [35]. Our results reveal higher rate of PONV after LSG. Fluid management of patients in our study group was comparable, although it slightly favored LRYGB group. Patients who underwent LRYGB less frequently required intravenous fluid administration during the operation day and more often preserved high oral fluid intake during the hospitalization. LRYGB group also achieved higher urination volume in postoperative course. One of the factors that determine the decision to discharge a patient after surgery, according to the ERAS protocol, is a requirement concerning administration of medications. Diuretic and painkiller administration was not influenced by the type of bariatric procedure. Prevalence of postoperative fever after LRYGB was significantly higher than after LSG in our study group. Postoperative fever is one of the most consistent signs for leakage after bariatric surgery, although we did not observe those relations in a study group [36, 37].

Mean LOS was slightly higher among patients in LSG group, in opposition to several previous publications, which included patients who were undergoing non-ERAS perioperative care [26, 27]. Studies by Young et al. and Albeladi et al. show no significant differences in LOS between LSG and LRYGB patients [22, 24]. We believe that prolonged LOS among patients undergoing ERAS protocol after LSG is a result of higher incidence of PONV and lower oral fluid intake, which are both associated with worse functional recovery.

Readmission may be more likely to occur within the first few weeks after surgery for LSG patients compared to LRYGB patients; nevertheless, LRYGB should be followed closely within the first 3 months to manage potential complications that would require readmission [38]. There was no statistically significant difference in incidence of readmissions among our patients.

### Limitations of the Study

The limitations of the present study are typical for non-randomized design and relatively small group. Influence of the type of procedure on excess weight loss and improvement of obesity-related comorbidities after bariatric treatment was not analyzed.

## Conclusion

There are significant differences in the course of postoperative care conducted accordingly with ERAS protocol among patients treated with LRYGB and LSG. Postoperative treatment after LSG requires significantly more supervision and longer time until functional recovery is achieved.
